# Massive ascites due to lupus peritonitis in a patient with pre-eclampsia and systemic lupus erythematosus: a case report

**DOI:** 10.1186/s12884-022-04550-0

**Published:** 2022-03-14

**Authors:** Shunya Sugai, Kazuaki Suda, Kana Tamegai, Kazufumi Haino, Takeshi Nakatsue, Ichiei Narita, Takayuki Enomoto, Koji Nishijima

**Affiliations:** 1 Department of Obstetrics and Gynecology, Niigata University Medical and Dental Hospital, 1-754 Asahimachi-dori, Chuo-ku, Niigata, 951-8520 Japan; 2 Division of Clinical Nephrology and Rheumatology, Niigata University Graduate School of Medical and Dental Sciences, Niigata, Japan; 3 Department of Obstetrics and Gynecology, Niigata University Graduate School of Medical and Dental Sciences, Niigata, Japan

**Keywords:** Ascites, Peritonitis, Pre-eclampsia, Systemic lupus erythematosus

## Abstract

**Background:**

Patients with systemic lupus erythematosus (SLE) are associated with pre-eclampsia. Pre-eclampsia can have systemic manifestations, such as ascites. Lupus peritonitis, a rare condition in patients with SLE, can also cause ascites.

**Case presentation:**

A 31-year-old woman, primigravida, with SLE had a blood pressure of 170/110 mmHg and proteinuria at 29 weeks of gestation. She was diagnosed with pre-eclampsia. Her blood pressure was stabilized by an antihypertensive drug. At 30 weeks of gestation, a cesarean section was performed for maternal safety because of decreased urine output and massive ascites. Postoperatively, re-accumulation of ascites was observed. On the fourth postoperative day, ascites (approximately 3 L) was discharged from the cesarean section wound. A decrease in serum complement concentrations was observed, and she was diagnosed as having lupus peritonitis. The steroid dose was increased and she recovered well thereafter.

**Conclusions:**

Ascites occurs in pre-eclampsia and SLE, but determining which of these conditions causes ascites can be difficult. However, careful observation is necessary because of the differences in treatment of these two conditions.

## Background

Systemic lupus erythematosus (SLE) is a well-known autoimmune disease with systemic inflammatory symptoms. SLE is often associated with pregnancy because it is more common in women of childbearing age. Pregnant women with SLE have an increased risk in the perinatal period, including a higher risk of pre-eclampsia [1]. Pre-eclampsia occurs in 3–5% of healthy pregnant women, but in patients with SLE, the complication rate is as high as 25% [2]. Additionally, SLE with pre-eclampsia is associated with an increased risk of preterm birth, intrauterine growth retardation, and low birth weight compared with SLE without pre-eclampsia [3]. The exact etiology of pre-eclampsia has not been determined, but it is a multisystem disorder that is unique to pregnant women, with a variety of clinical manifestations.

Ascites is one of the clinical manifestations of pre-eclampsia. The most probable cause of ascites is systemic capillary leakage due to dysfunctional vascular endothelial cells and decreased intravascular pressure [4]. In SLE, ascites can also be present as due to lupus peritonitis [5].

We report a rare case of ascites in an SLE patient who was diagnosed with pre-eclampsia and lupus peritonitis was strongly considered as the cause of massive ascites.

## Case presentation

The patient was a 31-year-old Japanese woman who was gravida 3, para 0. She had been diagnosed with SLE in accordance with the American College of Rheumatology criteria 8 years previously because of malar rash, discoid rash, photosensitivity, and positive antinuclear and antiphospholipid antibodies. She also had complications of lupus nephritis. After being diagnosed, she experienced two spontaneous miscarriages. Since 2019, her medications (prednisolone 5 mg/day, tacrolimus hydrate 3 mg/day, and aspirin 100 mg/day) remained unchanged for 20 months as her disease was stable. She had a spontaneous pregnancy, and then heparin therapy was added to prevent miscarriage. At each prenatal checkup, we checked her blood pressure, urine test results, and fetal growth every 2 weeks. She also had anti-Ro antibodies and was evaluated for fetal heart block with serial fetal echocardiography starting at 16 weeks of gestation. The progress of her pregnancy was uneventful.

At 25 weeks of gestation, her blood pressure was elevated (130–140/80–90 mmHg), and her spot urine protein to creatinine (P/C) ratio was 0.1 mg/mg. The complement 3 (C3) concentration was slightly low at 68.1 mg/dL (normal range: 73.0–138.0 mg/dL), and C4 and CH50 concentrations were normal at 27.9 mg/dL (normal range: 11.0–31.0 mg/dL) and 34 U/mL (normal range: 30–46 U/mL), respectively. She was admitted at 29 weeks and 4 days of gestation because of pre-eclampsia, with a blood pressure of 170/110 mmHg.

After hospitalization, her blood pressure was stabilized by intravenous nicardipine; however, her spot urine P/C ratio worsened to 11.8 mg/mg, and urine output had decreased to 600 mL/day. The estimated fetal weight was 1235 g (− 0.9 standard deviation). She was administered two doses of betamethasone (12 mg) at an interval of 24 h to achieve fetal lung maturity. Intensive fetal surveillance was frequently performed by cardiotocography and ultrasonography.

**Fig. 1:**
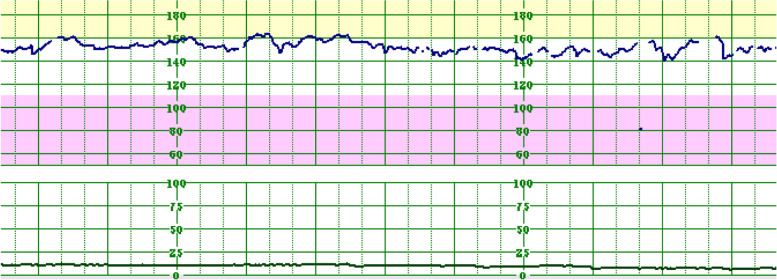
Cardiotocography showing a fetal heart rate of 150 bpm, accelerations, moderate variability, and no deceleration

**Fig. 2:**
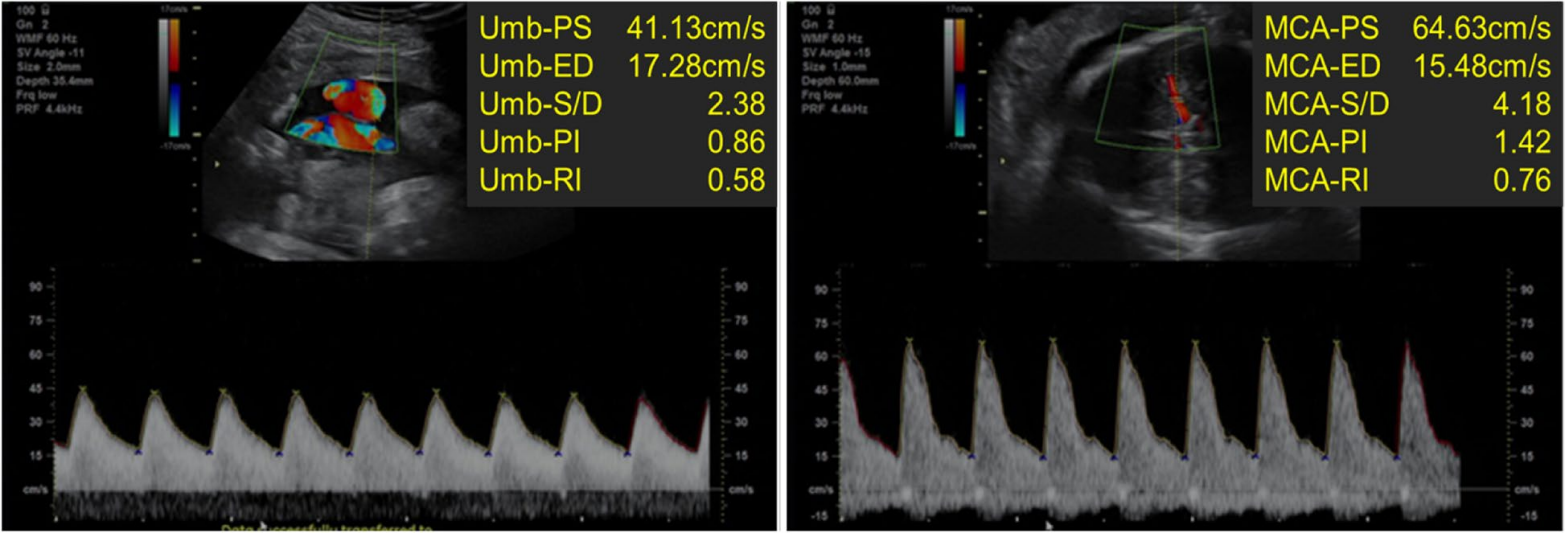
Doppler ultrasonography. The pulsatility index (PI) and resistance index (RI) of the umbilical artery (Umb) are within the normal ranges. The PI of the fetal middle cerebral artery (MCA) is at the lower limit of normal (5th percentile)

On the fifth day of admission, the fetal monitoring remained satisfactory on the basis of cardiotocography **(**Fig. 1**)** and Doppler ultrasonography **(**Fig. 2**)**. However, the maternal condition had deteriorated as evidenced by a further increase in the spot urine P/C ratio (14.9 mg/mg), decreased urine output to 272 mL/day, decreasing complement levels (C3: 59.2 mg/dL, C4: 14.9 mg/dL, and CH50: 18 U/mL), and a new finding of ascites during ultrasonography. The next day, she complained of increasing abdominal distension associated with vomiting and diarrhea. Clinically and by ultrasonography, her ascites had worsened. A decision was then made to terminate the pregnancy via an emergency cesarean section. Intra-operatively, there was massive ascites, and 900 mL of fluid was removed, with no other abnormalities noted. The delivery was uneventful, and she delivered a female neonate with Apgar scores of 7 and 8 at 1 and 5 min, respectively.

**Fig. 3:**
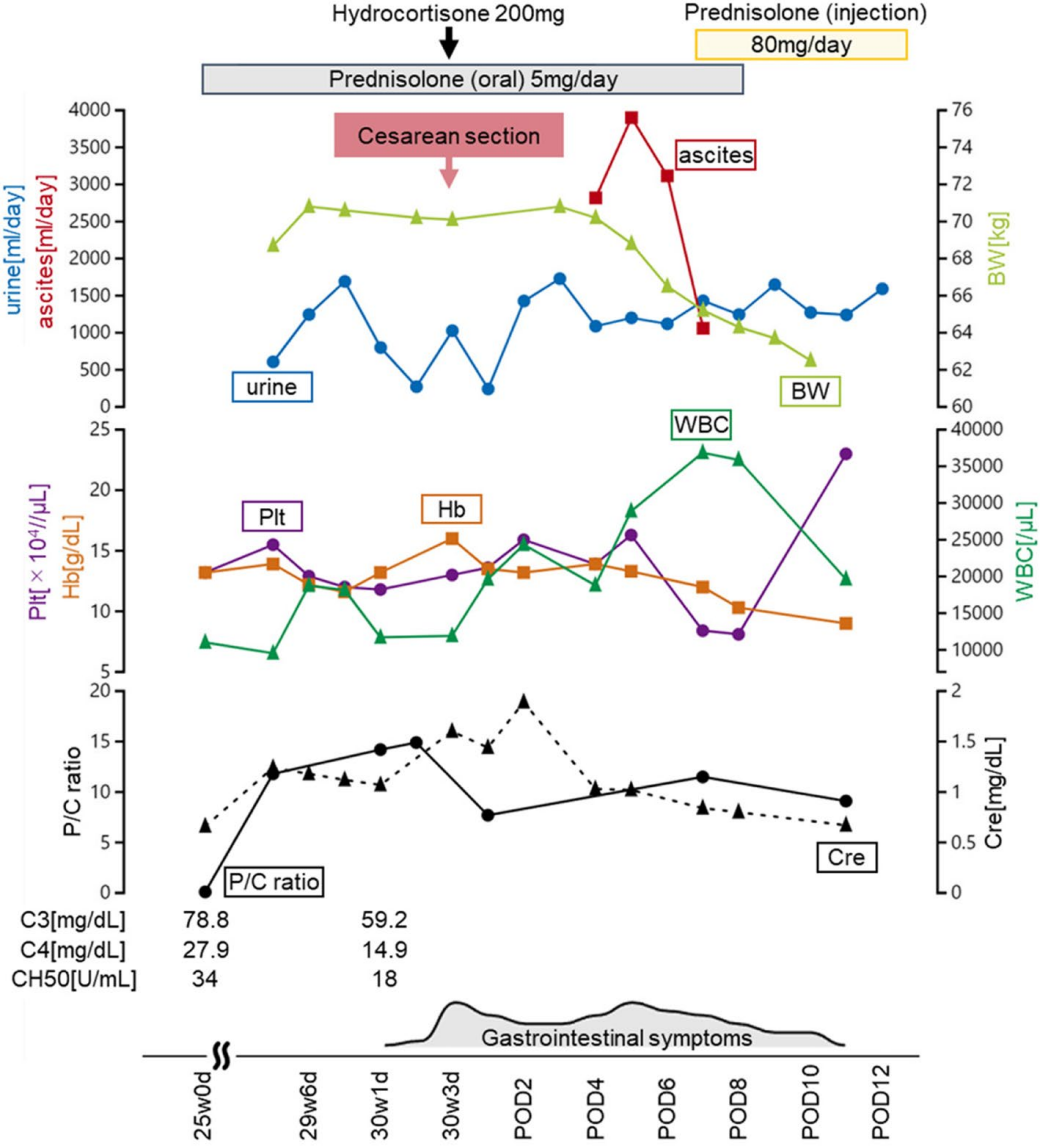
The patient’s clinical course. BW: body weight, Cre: creatinine, C3/4: complement 3/4, CH50: 50% hemolytic complement, Hb: hemoglobin, P/C ratio: spot urine protein to creatinine ratio, Plt: platelets, POD: postoperative day, WBC: white blood cells

Postoperatively, the reduced urine output persisted, and her abdomen became distended again clinically, which was confirmed ultrasonographically as re-accumulation of intraperitoneal fluid. The problem worsened and led to seepage of yellow-colored fluid through the cesarean wound. Ureteric or bladder injury was ruled out by a negative indigo-carmine dye test. She continued to experience vomiting and diarrhea. We then considered the possibility of lupus peritonitis and decided to intensify the immunosuppressive treatment. Intravenous prednisolone (80 mg) was administered, and the seepage of fluid from the cesarean wound ceased. Ultrasonography 5 days later confirmed a small amount of ascites confined to the pouch of Douglas. The patient’s clinical course is shown in Fig. 3.

Prednisolone was changed to oral administration, and the dose was tapered. Antihypertensive agents were also adjusted. Decreased urine protein concentration was also observed. She was discharged 6 weeks after surgery. Her neonate remained in the neonatal intensive care unit for 9 weeks and was discharged in a stable condition.

## Discussion and conclusions

The findings in this case suggested that the effects of lupus peritonitis should be considered if pregnant women with SLE who develop pre-eclampsia have massive ascites.

SLE is more common in women, with a female-to-male ratio of 9:1 [6]. SLE is mainly found in women of childbearing age, and pregnancy is considered high-risk owing to the associated maternal and perinatal morbidities, such as preterm birth, thrombosis, infection, and mortality [1].

Pre-eclampsia is a specific complication of pregnancy that is characterized by elevated blood pressure with proteinuria and/or organ dysfunction [7]. The definitive treatment for pre-eclampsia is termination of pregnancy. Ascites can be encountered in pre-eclampsia and has been reported in 21% of cases. The presence of maternal ascites is independently associated with adverse maternal events [8].

In SLE, lupus peritonitis may present with ascites and gastrointestinal symptoms, such as abdominal pain, diarrhea, bloating, nausea, and anorexia [5]. Ascites is thought to be caused by vasculitis of the peritoneum, and edema with intestinal swelling contribute to the symptoms [9]. These complications are signs of disease flare-up [10]. In this patient, the problem was further complicated by the development of pre-eclampsia, which can also cause ascites.

In this patient, ascites associated with pre-eclampsia was the first possible diagnosis that we considered. If the ascites was caused by pre-eclampsia, it should have resolved with termination of pregnancy. However, even after the termination of pregnancy, the ascites continued to worsen. Complement concentrations were decreased, which suggested that the SLE had worsened. Additionally, the patient had gastrointestinal symptoms, such as diarrhea, abdominal distension, and nausea, which suggested a high probability of lupus peritonitis. We speculate that the patient probably had edema and swelling of the intestine.

The treatment for pre-eclampsia is termination of pregnancy and that for SLE is immunotherapy, and an accurate diagnosis is important because the treatments are different. However, sometimes this is not possible because blood and urine tests lack specific findings, and the two conditions may overlap. In clinical practice, we believe that the first priority is to terminate the pregnancy for maternal safety. Immunotherapy then needs to be intensified, and generally involves steroids [5]. The administration methods are high-dose or pulse therapy. In this case, the patient responded well to high-dose steroids.

In developing countries, the likelihood of severe SLE is higher than that in developed countries because of the poor quality of care and access to care [11]. SLE is more likely to flare during pregnancy and during the first 3 months postpartum [12]. Therefore, intensifying immunotherapy in advance may be useful, especially in developing countries.

In conclusion, the presence of massive ascites in a pregnant woman with SLE who has developed pre-eclampsia may be due not only to the effects of pre-eclampsia, but also to those of lupus peritonitis.

## Data Availability

All data related to this report are available from the corresponding author on reasonable request.

## Abbreviations

P/C ratio: Protein to creatinine ratio
SLE: Systemic lupus erythematosus
C: Complement
